# Colchicine intolerance in FMF patients and primary obstacles for optimal dosing

**DOI:** 10.3906/sag-2001-261

**Published:** 2020-08-26

**Authors:** Hasan SATIŞ, Berkan ARMAĞAN, Erdal BODAKÇI, Nuh ATAŞ, Alper SARI, Nazife Şule YAŞAR BILGE, Dilek YAPAR, Reyhan BİLİCİ SALMAN, Gözde Kübra YARDIMCI, Hakan BABAOĞLU, Levent KILIÇ, Berna GÖKER, Şeminur HAZNEDAROĞLU, Timuçin KAŞİFOĞLU, Umut KALYONCU, Abdurrahman TUFAN

**Affiliations:** 1 Department of Internal Medicine, Faculty of Medicine, Gazi University, Ankara Turkey; 2 Department of Internal Medicine, Faculty of Medicine, Hacettepe University, Ankara Turkey; 3 Department of Internal Medicine, Faculty of Medicine, Osmangazi University, Eskişehir Turkey; 4 Department of Public Health and Biostatistics Faculty of Medicine, Gazi University, Ankara Turkey

**Keywords:** Colchicine, side effect, intolerance, toxicity, dose, familial Mediterranean fever

## Abstract

**Background/aim:**

Colchicine is the mainstay of treatment in FMF. However, in daily practice it is not easy to maintain effective colchicine doses in a substantial number of patients due to its side effects. In this study, we aimed to investigate prevalence and risk factors for colchicine side effects that limit optimal drug dosing and cause permanent discontinuation.

**Materials and methods:**

All patients were recruited from “FMF in Central Anatolia” (FiCA) cohort, 915 adults with a minimum follow-up time of 6 months during which they had obeyed all treatment instructions. Demographic and anthropometric data, FMF disease characteristics, disease severity, complications, and treatment features were recorded on a web-based registry. Prevalence of colchicine intolerance and characteristics of intolerant patients were analyzed.

**Results:**

Effective colchicine doses cannot be maintained in 172 (18.7%) subjects. Main side effects that limit optimal dosing were as follows: diarrhea in 99 (10.8%), elevation in transaminases in 54 (5.9%), leukopenia in 10 (%1.1), renal impairment in 14 (1.3%), myopathy in five (0.5%), and allergic skin reaction in two. Colchicine had to be permanently ceased in 18 (2%) patients because of serious toxicity. Male sex and obesity were found to be associated with liver toxicity, and having a normal body weight was associated with diarrhea. Chronic inflammation and proteinuria were more common in colchicine-intolerant patients, and they had reported more frequent attacks compared to those tolerating optimal doses.

**Conclusion:**

Colchicine intolerance is an important problem in daily clinical practice, mainly due to diarrhea and liver toxicity. Suboptimal colchicine dosing is associated with complications.

## 1. Introduction

Familial Mediterranean fever (FMF) is the most frequent autoinflammatory disease (AID), which is characterized by self-limited episodes of fever, polyserositis, arthritis and/or erysipelas, like skin rash [1]. FMF is more prevalent in individuals from Mediterranean populations, especially Turks, Arabs, non-Ashkenazi Jews, and Armenians, while there are several hundreds of patients described in non-Mediterranean countries. FMF attacks generally last for 1–3 days and impair quality of life. Moreover, some patients may develop secondary amyloidosis and renal failure as a consequence of chronic ongoing inflammation [2]. Amyloidosis is an irreversible life- and organ-threatening complication with reported prevalence of approximately 10% in FMF patients [3]. 

Colchicine is the mainstay of treatment because of its proven efficacy in reducing frequency, severity, and duration of attacks and development of secondary amyloidosis [4–6]. Colchicine is the only drug that prevents the development of amyloidosis with a minimal recommended dose of 1 mg/day [2]. Amyloidosis is observed almost invariably in those patients who do not use colchicine regularly or those with delayed diagnosis. Risk of amyloidosis is about 1% when colchicine is used regularly and as high as 50% without life-time colchicine prophylaxis. About 5–10% of patients do not respond well to colchicine in terms of controlling occurrence of debilitating attacks, but colchicine still seems to be protective against the development of amyloidosis. Hence, life-time colchicine treatment is mandatory in patients with FMF, unless a severe toxicity develops [7]. 

Colchicine has been used for centuries in the treatment of gout and is a well-known agent with a narrow therapeutic index. In appropriate dosages, colchicine is an effective and safe drug. The recommended dose of colchicine is 1–2.5 mg/day in treatment of FMF; however, despite dose reduction, a significant proportion of patients cannot tolerate prescribed doses and continue to suffer from attacks. Recently, antiinterleukin-1 (anti-IL-1) treatment has made a breakthrough in the treatment of colchicine-resistant patients. However, long-term efficacy and safety of anti-IL-1 antagonists have not been established, and their high costs limit their extended use. 

The side effects of colchicine are well-defined. The most common side effects are gastrointestinal intolerance, diarrhea, nausea, and vomiting in particular [8]. Chronic colchicine use may cause blood cytopenia, liver dysfunction, and myopathy [9], especially when combined with other drugs, such as statins, cyclosporine, and clarithromycin [10,11]. Although the side effects of colchicine are well-known, the prevalence and risk factors of its side effects in FMF have not been studied in any systematic study. Herein, we aimed to investigate the causes of colchicine intolerance, obstacles in the determination of the optimal colchicine dosage and the disease course of colchicine-intolerant patients. 

## 2. Materials and methods

### 2.1. Patients and study design

All patients were recruited from FMF in Central Anatolia (FiCA) cohort, which is a duplication-disabled, internally and externally controlled, cross-sectional, multicenter accessible web-based cohort. FiCA cohort comprised adult FMF patients who were followed at outpatient rheumatology clinics of three university hospitals located in central Turkey and that receive referrals from the whole county. The study was conducted between January 2018 and December 2018. The diagnosis of FMF was made using Tel-Hashomer criteria [12]. The study protocol received institutional review board approval (Date: 25.12.2017, approval number 2017-622), and all the participants gave written informed consent in the format required by the relevant authorities and/or boards. The study was conducted in compliance with Institutional Review Board/Human Subjects Research Committee requirements. 

Demographic and anthropometric data, FMF disease features, comorbid conditions (asthma, fibromyalgia, diabetes, hypertension, thyroid disease, chronic kidney/liver disease, rheumatological disease), and concomitant medication usage (antidepressant, antihypertension, antidiabetic, steroid and nonsteroid antiinflammatory drugs, mycophenolate mofetil, azathioprine, methotrexate, leflunomide, sulfasalazine) other than colchicine disease complications were meticulously questioned. Laboratory investigations and genotype data (if available) were recruited from computer-based health records. FMF disease severity was assessed by International Severity Score for FMF (ISSF) [13], and FMF-related damage was assessed by autoinflammatory disease damage index (ADDI) [14]. Persistent chronic inflammation was defined as increased C-reactive protein levels above the normal limits measured in attack-free periods at least ≥2 weeks apart and must be present ≥75% of follow-up visits. 

### 2.2. Treatment characteristics and side effects

Used medications along with their doses, colchicine adherence, and side effects were carefully questioned. Colchicine intolerance is described as not being able to increase the dose of colchicine to effective levels to prevent the occurrence of FMF attacks to “less than one attack in a 6-month period” and to normalize APR. Minimal maintenance dosage of colchicine was described as 1 mg/day, which is required for the prevention of amyloidosis, and dose increase was allowed up to 2.5 mg per day, which is the recommended maximal safe dose [2].

Side effects that were accepted clinically significant were as follows: diarrhea (passing loose stools three or more times a day), abnormality in liver function tests [elevation of transaminases more than 2.5 fold of the upper limit of normal (ULN) (for permanent discontinuation elevation of 2.5xULN in 5 consecutive visits)]; myopathy [objective muscle weakness or elevation of a creatine kinase (CK) level of 3xULN for more than 3 times [15]], and leukopenia (absolute neutrophil number less than 1500/mm3). Oligospermia is described according to WHO criteria [16] and further assessed in case of patient declaration. 

Finally, prevalence of all colchicine side effects was presented, and risk factors for their occurrence were assessed. Moreover, disease-related damage and complications between groups were compared. 

### 2.3. Statistical analysis

Statistical analyses were performed by SPSS software v15.0 (Chicago, IL). Categorical variables were presented as numbers and percentages, and continuous variables were presented as mean ± standard deviation (SD) or median (interquartile range: IQR), respectively, according to their normality of distribution. The conformity of continuous variables to normal distribution was evaluated by using visual (histogram and probability graphs) and analytical methods (the Kolmogorov–Smirnov/Shapiro–Wilk tests). In cases where continuous variables were normally distributed, Student’s t-test was used; and in other cases, the Mann–Whitney U test was used for comparisons. 

For the determination of the underlying risk factors for the occurrence of a single side effect, we assessed the relationship between each variable and that particular side effect by comparing their features with tolerant patients. Variables with significant association with a particular side effect were then entered into binary logistic regression analysis, and those that remained significant were retained in the final model. Age, sex, comorbidities (present or not), concomitant medications (present or not), literacy, smoking, and obesity were included in the analyses. Hosmer–Lemeshow goodness-of-fit statistics were used to assess model fit. A 5% type I error level was used to infer statistical significance. 

## 3. Results

Original FiCA cohort comprised 971 patients, but 56 patients were excluded from the current study due to treatment incompliance. Remaining 915 FMF patients who had been followed up for ≥6 months were included in the analysis. The demographic, clinical, and disease characteristics of patients are summarized in Table 1. Genetic test results were available for 761 patients, and common variants are presented in Table 2. 

**Table 1:** Clinical and treatment characteristics of colchicine-tolerant and -intolerant groups.

	Colchicine tolerant N = 743	Colchicine intolerant N = 172	P
Female n (%)	452 (60.8%)	109 (62.6%)	0.770
Age, years	36.1 ± 11.3	38.59 ± 12.7	0.017
Smoking n (%) Alcohol usage n (%)	221 (29.7%) 65 (8.7%)	38 ( 26.7%) 13 (7.6%)	0.573 0.739
Education year	11.73 ± 4.12	11.76 ± 4.13	0.600
Age at FMF onset, years	13.71 ± 10.2	14.22 ± 11.65	0.790
FMF disease duration, years	22.43 ± 11.58	24.37 ± 13.21	0.054
Treatment duration, years	11.67 ± 7.89	12.01 ± 8.99	0.510
Number of mutations n (%) Mutation negative Single allelic mutation Biallelic mutation	52 (7%) 235 (41.4%) 332 (58.5%)	8 (5.6%) 60 (42.5%) 81 (57.4%)	0.950
Disease severity n (%) Mild Moderate Severe	383 (51.5%) 324 (43.6%) 36 (4.8%)	61 (35.3%) 84 (48.8%) 27 (15.6%)	0.001
Obesity	11.4%	10.6%	0.893
Comorbid disease* n (%) Hypertension Chronic kidney disease	179 (24.1%) 71( 9.6%) 7 (0.9%)	63 (36.6%) 27( 15.7%) 20 (11.6%)	0.0010.028<0.001
Concomitant medication n (%)	181 (24.4%)	52 (30.2%)	0.538

*Other than hypertension and chronic kidney disease, the frequency of other comorbidities was similar.

**Table 2:** Frequency of the ten most common MEFV gene variants in the study population.

MEFV gene variants	n (%)
M694V/M694V	215 (28)
M694V/-	98 (13)
M694V/M680I	75 (9.7)
M694V/V726A	49 (6.1)
M694V/E148Q	37 (4.5)
M680I /M680I	24 (3.2)
M680I /-	21 (3.1)
V726A/-	23 (2.8)
M680I /V726A	20 (2.4)
M694V/R761H	13 (1.8)

Among all patients, 172 of them had colchicine intolerance (18.8%). Median used dose of colchicine in the tolerant group was 1 (1–2) mg, whereas it was 1 (1–1) mg in the intolerant group (P < 0.001). The most common side effects causing colchicine intolerance were diarrhea in 99 (9.7%) and liver toxicity in 54 patients (5.3%). Leukopenia (n=10), myopathy (n=5), nausea (n=4), and skin rash (n=2) were other reasons for the drug intolerance (Table 3). 

**Table 3:** Prevalence of all side effects of colchicine and reasons for drug discontinuation.

Side effect (n)	All side effects N = 172*	Permanent cessation N = 18*
Diarrhea	99	11
Liver toxicity	54	4
Leukopenia	10	1
Muscle toxicity	5	2
Skin reaction	2	-
Nausea	4	-
Infertility	2	-

* some patients had more than one clinically significant side effect

In 18 patients (1.9%), because of the serious side effects, colchicine treatment had to be permanently discontinued. These patients did not tolerate even 0.5 mg/day doses of colchicine and were treated with interluekin-1 antagonists. Intractable diarrhea was the leading reason for permanent discontinuation in 12 (1.1%) patients, followed by liver toxicity, which occurred in four patients. Myopathy was observed in two patients because of severe CK elevation, which was completely resolved after discontinuation. Male infertility due to oligospermia was evident in two patients. 

Diarrhea was evident in 99 patients and was more prevalent in those with body mass index (BMI) of <25 kg/m2 and in women. In multivariate regression analysis, having normal BMI was found to be an independent risk factor for diarrhea (OR: 3.57 95%CI: 1.9–6.7). Fifty-four patients had developed liver toxicity because of elevation in transaminases. Liver toxicity was more common in those with BMI ≥ 25 kg/m2, males and increasing age. However, male sex and obesity were found to be the independent risk factors for liver toxicity (OR: 2.25, 95%CI: 1.12–4.52 and OR: 2.39, 95%CI: 1.08–5.30), respectively.  

Colchicine-intolerant patients had a worse disease course than colchicine-tolerant patients (Table 4). Intolerant patients had more attacks, persistent inflammation, comorbidity, proteinuria, amyloidosis, and worse ADDI and ISSF scores compared to the colchicine-tolerant group.  

**Table 4:** Disease course in colchicine-tolerant and -intolerant patients.

	Colchicine Tolerant N = 743	Colchicine Intolerant N = 172	P-value
Chronic inflammation n (%)	115 (15.4%)	45 (26.1%)	<0.001
Number of attacks in the last year (median) (IQR)	2 (5)	4 (8)	<0.001
Proteinuria n (%)	44(5.9 %)	20 (11.6%)	0.025
Amyloidosis n (%)	33 (% 4.4)	23 (13.3%)	<0.001
ADDI (median) (IQR)	1 (1)	1 (1)	<0.001

ADDI: autoinflammatory disease damage index, FMF: familial Mediterranean fever

## 4. Discussion

In this multicenter, cross-sectional study, prevalence of colchicine intolerance and risk factors for colchicine side effects were investigated. Our study was the first study to show the real-life experience related to colchicine intolerance. Moreover, it was observed that colchicine intolerance hampered management of FMF and increased complications of diseases, such as amyloidosis, as a result of suboptimal dosing. 

Colchicine is the mainstay of treatment of FMF due to its proven efficacy in prevention of attacks and amyloidosis even in nonresponsive patients in terms of occurrence of attacks. Therefore, life-time colchicine prophylaxis is warranted, unless a severe toxicity develops. Colchicine intolerant patients are not considered “colchicine resistant” in EULAR recommendations, and treatment of these patients is not as clear as it is in colchicine-resistant patients. Our study has shown that these patients are suboptimally managed as evidenced by ongoing attacks and persistently-elevated APRs. About one-third of FMF patients had persistent elevation of APR, though they did not report that typical attacks predisposed them to secondary amyloidosis [17]. Moreover, colchicine compliance and M694V homozygous mutation are two main factors related to subclinical inflammation and amyloidosis [18,19]. 

There were more patients with elevated APRs and damage including proteinuria and amyloidosis in the colchicine-intolerant group. Hence, interventions like adding antimotility drugs for diarrhea and use of IL-1 antagonists to control persistent inflammation in clinical practice may prevent them from disease complications. In our study, the colchicine-intolerant group tended to have a longer disease duration (P = 0.054), and it is well known that drug compliance rates drop in chronic illnesses. Even minimal side effects might reduce patients’ compliance to treatment [20]. Because it is a life-long disease and because of the possibility of break in compliance, FMF patients should be frequently warned about the regular use of effective doses of colchicine. 

There is limited data in the literature about colchicine intolerance. A few studies reported a compliance ratio of 58–64% to colchicine [21,22]; moreover, so far, no study has investigated the underlying factors that increase the rate of intolerance. Diarrhea is among the most commonly observed side effects, affecting approximately 20% of patients [23]. This side effect can be handled by dividing the daily dose or use of antimotility agents [2]. Previously, several mechanisms were proposed for gastrointestinal intolerance, especially diarrhea, including drug interactions [24,25]. In a recent study, 12 FMF patients were followed prospectively. Three of them had steatorrhea, and their jejunal biopsy showed decreased Na+-K+ adenosine triphosphatase (Na+-K+ ATPase) activity, which was claimed to be responsible for colchicine toxicity [26]. Also, some of the intestinal enzyme activities, such as lactase, sucrase, and maltase, were decreased, which might cause diarrhea. Previously, antimotility agent use was recommended in patients with diarrhea [27]. In our cohort, antimotility agents worked in 12 of 19 patients, who were able to reinstitute colchicine, but these agents were not tested in all diarrheic patients. Interestingly, having normal BMI was found to be associated with diarrhea in our study. Colchicine is a lipophilic molecule; and in overweight-obese patients, the volume of distribution might be different than normal weight population. At the same oral dose, altered pharmacokinetics of colchicine due to obesity may explain the low prevalence of diarrhea.

Liver toxicity is one of the common side effects of colchicine that cause intolerance. Colchicine was mainly metabolized by the liver [25] and excreted by biliary tract entering in enterohepatic pathway [25]. Although elevation of transaminases is a well-known side effect of colchicine, there are no data on the mechanism of toxicity and facilitating factors that increase hepatic injury. In our study, obesity and male sex were found to be associated with increased liver toxicity. Nearly all obese patients had fatty liver, and adding a potential hepatotoxic drug might increase liver toxicity. Colchicine was metabolized by CYP3A4 enzymes in the liver [28]. It was shown that CYP3A4 enzyme activity was higher in females compared to males as demonstrated by higher protein and mRNA expression [29], which may explain increased toxicity seen in males.

In the literature, data on colchicine-related male infertility were inconsistent [30, 31]. Colchicine concentration in sperm’s microtubule was 3.000-fold higher than plasma concentrations [32]. In our study, two male patients suffered from azoospermia, which was restored to normal numbers after cessation with similar reported cases in the literature [31,33].

Use of IL-1 antagonists were recommended by EULAR in colchicine-intolerant patients [2]. In our study, nine patients were given IL-1 antagonists due to severe side effects of colchicine and frequent attacks. Of these, one patient developed injection site reaction to anakinra and switched to canakinumab, and the others continued with anakinra treatment. In the follow up, eight of nine patients went into remission as it was evident in the absence of attacks, improved life quality and normalization of APR levels. Hence, IL-1 antagonists seem to be effective and well-tolerated in colchicine-intolerant patients, and we recommend the use of these drugs in those with elevated APR and frequent attacks. Although the long-term efficacy of IL-1 antagonists in the prevention of secondary amyloidosis has yet to be determined, sustained normalization of APR levels may be extrapolated as reduced risk for amyloidosis.

There are some limitations to this study. Since the study was not prospective, temporal changes in colchicine tolerance might not be correctly estimated. Also, diarrhea was not evaluated with objective methods and was primarily based on patient declaration. Patients’ perception of diarrhea might be different and could be under- or overexpressed by patients. Yet another limitation is that some side effects might differ in different ethnic groups or geographic regions, but our study included subjects from the same ethnic group. Also, our study only comprised adult patients and might not reflect colchicine tolerance of children. 

In conclusion, colchicine intolerance is an important domain in the management of FMF patients. Suboptimal colchicine dosing due to drug intolerance may yield development of disease complications due to inadequate control of attacks and chronic inflammation. Use of IL-1 antagonists in such patients after careful evaluation of possible contributing factors to intolerance may decrease the development of future complications. 
